# Long-Term Outcomes of Implants Placed in Maxillary Sinus Floor Augmentation with Porous Fluorohydroxyapatite (Algipore^®^ FRIOS^®^) in Comparison with Anorganic Bovine Bone (Bio-Oss^®^) and Platelet Rich Plasma (PRP): A Retrospective Study

**DOI:** 10.3390/jcm11092491

**Published:** 2022-04-28

**Authors:** Biagio Rapone, Alessio Danilo Inchingolo, Stefano Trasarti, Elisabetta Ferrara, Erda Qorri, Antonio Mancini, Nicola Montemurro, Antonio Scarano, Angelo Michele Inchingolo, Gianna Dipalma, Francesco Inchingolo

**Affiliations:** 1 Interdisciplinary Department of Medicine, University of Bari “Aldo Moro”, 70121 Bari, Italy; ad.inchingolo@libero.it (A.D.I.); dr.antonio.mancini@gmail.com (A.M.); angeloinchingolo@gmail.com (A.M.I.); giannadipalma@tiscali.it (G.D.); francesco.inchingolo@uniba.it (F.I.); 2 Department of European Studies Jean Monnet of Lugano, 6900 Lugano, Switzerland; 3 Complex Operative Unit of Odontostomatology, Hospital S.S. Annunziata, 66100 Chieti, Italy; igieneeprevenzione@gmail.com; 4 Dean Faculty of Medical Sciences, Albanian University, Bulevardi Zogu I, Tirana 1001, Albania; erda79@yahoo.com; 5 Department of Neurosurgery, Azienda Ospedaliera Universitaria Pisana (AOUP), University of Pisa, 56100 Pisa, Italy; montemurronic@hotmail.com; 6 Department of Oral Science, Nano and Biotechnology, CaSt-Met University of Chieti-Pescara, 66100 Chieti, Italy; ascarano@unich.it

**Keywords:** sinus floor augmentation, autogenous bone, piezosurgery, vestibular region, implant, jawbone reconstruction, biomaterials

## Abstract

Purpose: The objective of this retrospective study was to evaluate the long-term clinical outcomes of bone regeneration procedures using algae-derived plant hydroxyapatite (Algipore^®^ FRIOS^®^) compared with demineralized anorganic bovine bone (Bio-Oss^®^), in combination with autologous blood-derived PRP. Materials and Methods: Partially edentulous patients with severe atrophy of posterior maxillary treated by means of the split bone technique in a two-stage grafting procedures were observed for up to seven years after implants placement. After surgeries, the natural porous fluorohydroxyapatite (FHA) (Algipore^®^ FRIOS^®^; Group, *n* = 29) or anorganic bovine bone (Bio-Oss^®^ Group, *n* = 28) with autogenous bone in a 50:50 composite ratio with PRP, were administered in a 2.8-mm critical-size defect (CSD). Four months later, implants were placed at second-stage surgery. Results: A sample of fifty-seven consecutive patients who required sinus augmentation was included in the study, and 57 implants were placed. There was no drop out or loss of follow-up of any case. Clinical and radiographic examinations revealed a comparable pattern of newly formed bone in both groups after seven years of functional loading for implants placed after sinus augmentation using porous fluorohydroxyapatite and anorganic bovine bone. No significant difference in marginal bone loss was found around implants in both groups. Conclusions: The favorable implant outcomes suggest both biomaterials are suitable for sinus grafting in severely atrophic maxillae.

## 1. Introduction

The maxillary sinus floor augmentation technique is widely used in the treatment of resorbed posterior maxilla and remains a challenge in regenerative surgery [1,2,3]. The main objective of alveolar bone grafting surgery is to facilitate the natural regenerative process of bone and restore an optimal functional status via the synergistic combination of placing bone grafting materials, cells, and growth factors [2,4,5,6,7]. Among the different available augmentation materials, autogenous bone has long been considered the gold standard in bone grafting procedures due to its limited immunological reactions [5,7,8,9]. Although autologous bone grafts have excellent biologic and mechanical properties, the occurrence of significant graft resorption or their oral exposures, limited amount of donor bone tissue, morbidity at the donor site, limited availability, and risk of infection have been described [6,10,11,12]. In recent years, alternative approaches have been developed to supply the reported disadvantages of autologous bone, having good biocompatibility, degradability, and a porous three-dimensional structure that benefit from osteocunduction, osteoinduction, and osteogenesis [13,14]. Biomaterials with new levels of biofunctionality for supporting regeneration of bone tissue, are emerging as interesting alternative method, in a wide range of surgical procedures, to mimic the regulatory characteristics of natural extracellular matrices (ECMs) and ECM-bound growth factor. The local methods for enhancement of the alveolar bone height encompass the use of biological bone grafts, synthetic grafts, and delivery of growth factors [15,16]. Bone scaffolds have the advantage of possessing high ability to reproduce their biological microenvironments and sustain the growth of new tissue. Natural polymers have become a main source for the manufacturing of biodegradable matrices due to their similar characteristics of natural bone, ability to provide a proper biochemical environment, and induce cell adhesion and migration, proliferation and osteogenic differentiation [17,18,19,20,21]. The porous fluorohydroxyapatitic (FHA) biomaterial as shown promise as candidate in suitable biomaterial for sinus grafting in severely atrophic maxillae [22]. Algipore^®^ FRIOS^®^ is a biomaterial, vegetable-based hydroxyapatite. It is highly analogous to the hydroxyapatite of natural bone and is manufactured from lime-impregnated red marine algae (Corallina officinalis) [23]. The biomaterial is processed through phases involving the pyrolytic segmentation of the native algae and by hydrothermal transformation of calcium carbonate (CaCO_3_) into FHA [Ca5(PO4)3OHxF1 x]. The structural features of 3D porous particles have shown a hierarchical pore system containing particles with a mean diameter of pores 10 mm periodically septated (mean interval 30 mm) and interconnected by microperforations of 1–4 µm. Every pore is limited by one layer of small FHA crystallites with a size of 25–35 nm. The average pore volume decreased from 1.05 cm^3^/g to 0.93 while the surface area averages 50 m^2^/g [23]. Bio-Oss^®^ has often been used for maxillary sinus floor elevation. It consists of deproteinized sterilized bovine bone constituted by a 90% of calcium-deficient carbonate apatite and 10% porcine collagen (type-I) [24,25,26]. The combination of autogenous bone and bovine bone material has been examined in several histological studies. It has been shown that hydroxyapatite (BioOss^®^) supports cell viability and allow cell proliferation [25]. In addition, the effect of using platelet-rich plasma (PRP) has been studied in the implant surgery setting aiming to accelerate bone regeneration [27,28]. In vitro and in vivo studies have confirmed and demonstrated the role of platelets, mostly represented by platelet-rich plasma (PRP) and platelet-rich fibrin (PRF), in supporting tissue healing, promoting a bone regeneration process, bone homeostasis and vascularization for the treatment of bone defects [29]. Combined use of bio-functionalized scaffolds composed of platelet-rich plasma (PRP) promotes tissue regeneration mediated by the release of several growth factors (GFs) [27,29]. Platelet alpha-granules are rich in GFs such as platelet-derived growth factor, transforming growth factor-β, and vascular endothelial growth factor that act in tissue repair, activating fibroblasts and inducing the extracellular matrix synthesis and remodeling [29]. Our study was aimed to evaluate long-term clinical outcomes of sinus floor augmentation procedures using algae-derived plant hydroxyapatite (Algipore) compared with demineralized anorganic bovine bone (Bio-Oss Geistlich Pharma, Wolhusen, Switzerland), in combination with autologous blood-derived PRP.

## 2. Materials and Methods

This retrospective comparative clinical study included 57 partially edentulous maxillary adult patients (>18 years of age). The trial was conducted from April 2015 to January 2021. The study was conducted according to the guidelines of the Declaration of Helsinki and approved by the local Ethics Committee of Albania University, Tiran, Albania (Nr. 171 Prot.—Date: 18 June 2015). The study adhered to the Strengthening the Reporting of Observational Studies in Epidemiology (STROBE) checklist guidelines. The study was conducted in accordance with the Declaration of Helsinki. Informed consent was obtained from all patients. Edentulous patients older than 18 years with need of dental implant placement in the posterior maxilla, and having a maximum of 4 mm residual height of the alveolar ridge at either site of the maxilla, were eligible for the study. Exclusion criteria were (1) smoking, (2) history of systemic disease which may have an effect on bone turnover (3) pregnancy or nursing, (4) medication, pre-existent periodontal disease (5), bone or non-mineralized tissue metabolism (6), cognitive disorders (7), and allergies (8). Preoperative orthopantomograms, CBCT scans and postero-anterior oblique radiographs were performed for each patient to assess the height of the maxillary alveolar bone, the dimensions of the maxillary sinus and the antero-posterior relationship of the maxilla to the mandible, and provide a higher degree of predictability of implant placement. Patients were divided into two groups: in Group Algipore^®^ (*n* = 29), piezosurgery was used for osteotomy and PRP was administered. Original bone was augmented with 50% porous fluorohydroxyapatite (FHA) FRIOS Algipore^®^ biomaterial and 50% particulate autogenous bone; in Group Bio-Oss^®^ (*n* = 28), piezosurgery was used for osteotomy and PRP was administered. Original bone was augmented with 50% anorganic bovine bone (Bio-Oss^®^) plus 50% articulate autogenous bone (Bio-Oss^®^ group, *n* = 29), in combination with autologous blood-derived PRP. Implant placement was planned for 4 months after grafting and carried out according to the manufacturer’s protocols. 

### 2.1. Surgical Procedures

**Figure 1:**
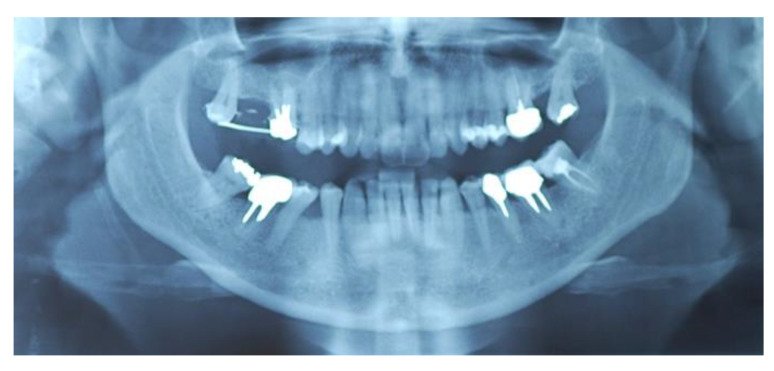
Preoperative panoramic X-ray (Group Algipore^®^).

**Figure 2:**
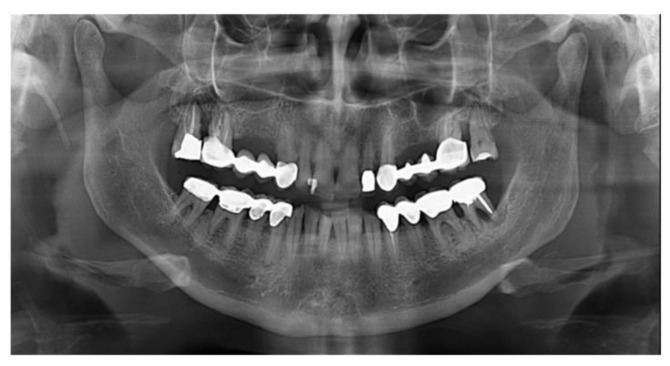
Preoperative panoramic X-ray (Group Bio-Oss^®^).

The split bone block technique and subsequent implant placement in a two-stage grafting procedure was performed. All implants were placed 4 months after sinus floor augmentation. Implant placement followed standard protocols according to the manufacturer’s instructions. The sinus floor augmentation procedure was performed with either a xenograft Bio-Oss^®^, 1–2 mm large granules, Geistlich Pharma AG, Wolhusen, Switzerland) OR Algipore^®^ (Friadent GmbH, Mannheim, Germany). To prevent surgical site and postoperative infections after the extraction, all patients received prophylactic antibiotic therapy: amoxicillin 500 mg (Zimox^®^—Pfizer Italia Srl; Latina, Italy) or clindamicyn (Zimox^®^—Pfizer Italia Srl; Latina, Italy) if allergic to penicillin was given twice a day, initiating 1 h prior to surgery and continued postoperatively for 4 days. In addition, rinsing for 60 sec with chlorhexidine (CHX) mouthwash 0.2% (Curasept DS–Curaden Healthcare S.p.A.; Saronno, Varese, Italy) prior to the surgery. After surgery, the patients were prescribed to rinse two times per day for 1 min for three weeks with 10 mL of CHX 0.2%. The baseline orthopantomogram showed a bony defect extending to the maxillary antrum (Figure 1 and Figure 2); the condition was subsequently confirmed by CT scan. 

**Figure 3:**
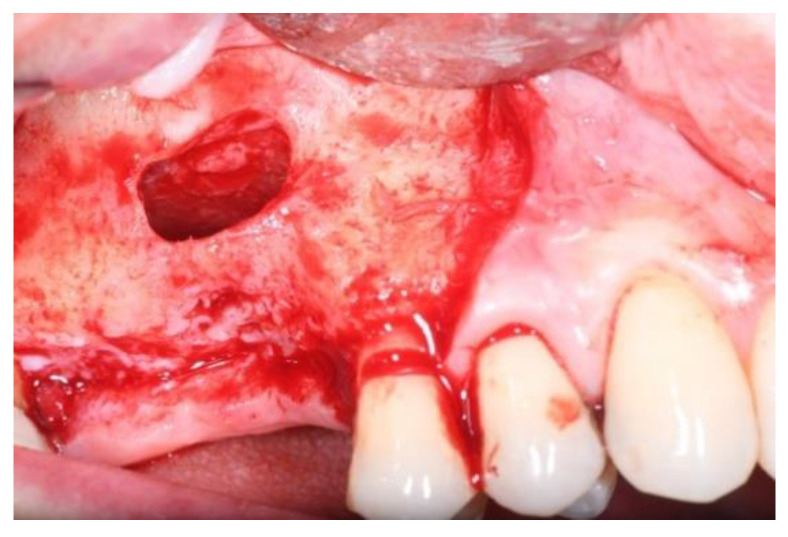
Group Algipore^®^.

**Figure 4:**
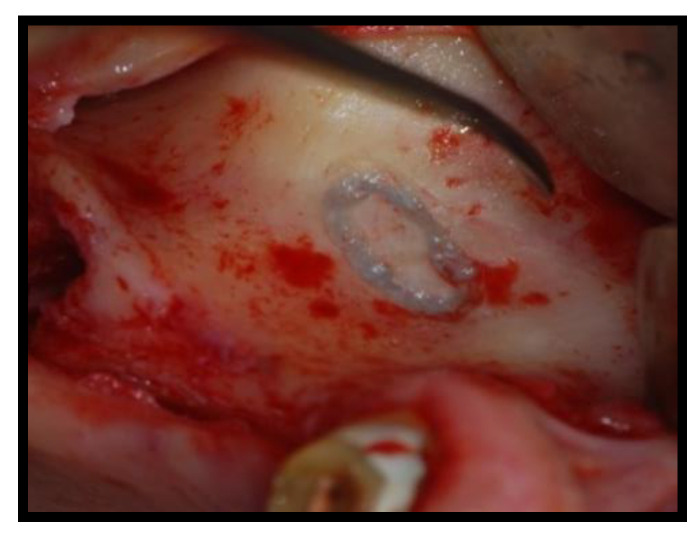
Group Bio-Oss^®^.

The sinus lift was performed using the particulate bone at 50% collected with scraper (META), consituted by Algipore^®^ FRIOS^®^ in the proportion 50% or Bio-Oss^®^ at 50% and 50% particulate autogenous bone (Figure 3 and Figure 4), in combination with autologous blood-derived PRP.

**Figure 5:**
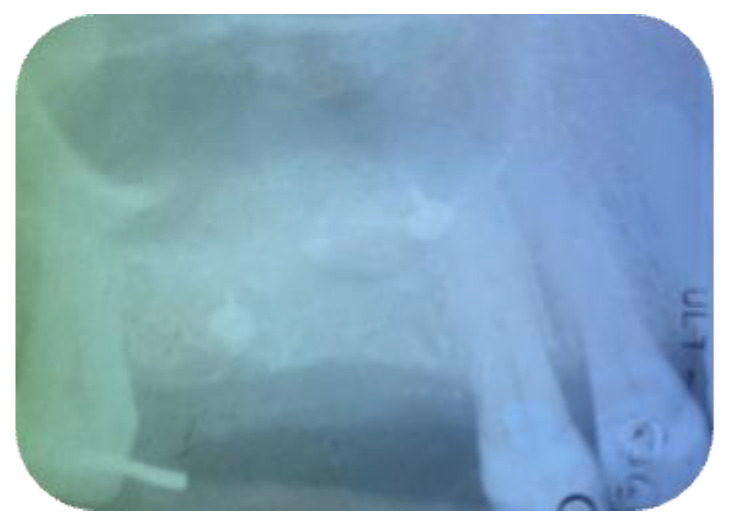
Rx after the sinus lift (Algipore^®^ Group).

**Figure 6:**
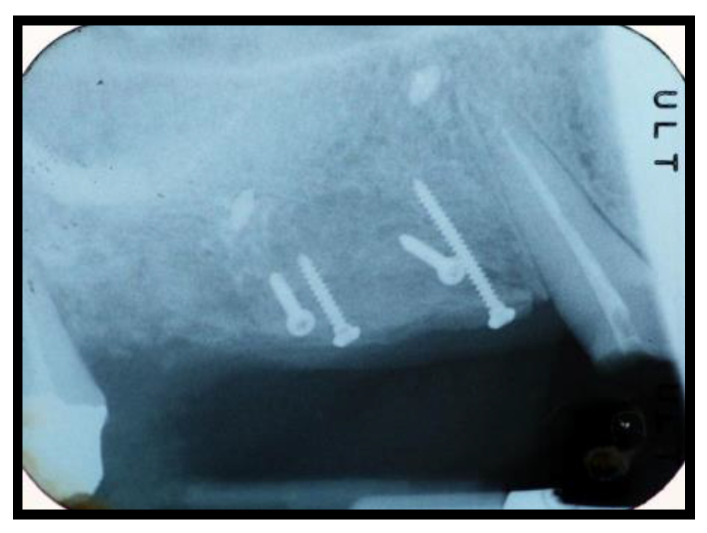
Rx after the sinus lift (Bio-Oss^®^ Group).

The large residual gap was filled with PRP activated with calcium chloride that allows platelet degranulation in order to obtain PRF (platelet-rich-fibrin) which was mixed with Bio Oss^®^. Before surgery, 36 mL of blood was collected and centrifuged at 1000 rpm for 20 min (which centrifuge), obtaining 4 mL of PRP from each. Sinus grafting was performed with an injection of liquid PRP and insertion of the PRF membrane. The distance between the implant collars and cortical was filled with a combination of Algipore^®^ FRIOS^®^ (Algipore^®^ Group) or Bio-Oss^®^ (Bio-Oss^®^ Group), Figure 5 and Figure 6. 

**Figure 7:**
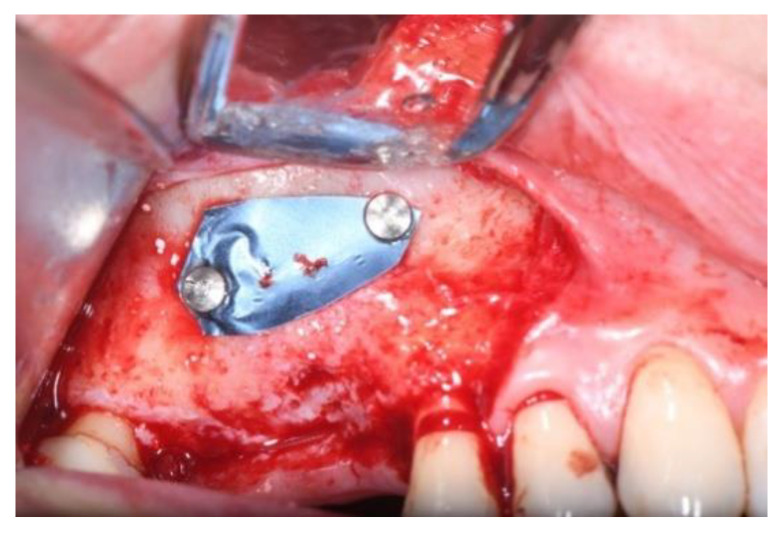
The titanium membrane placement (Group Algipore^®^).

**Figure 8:**
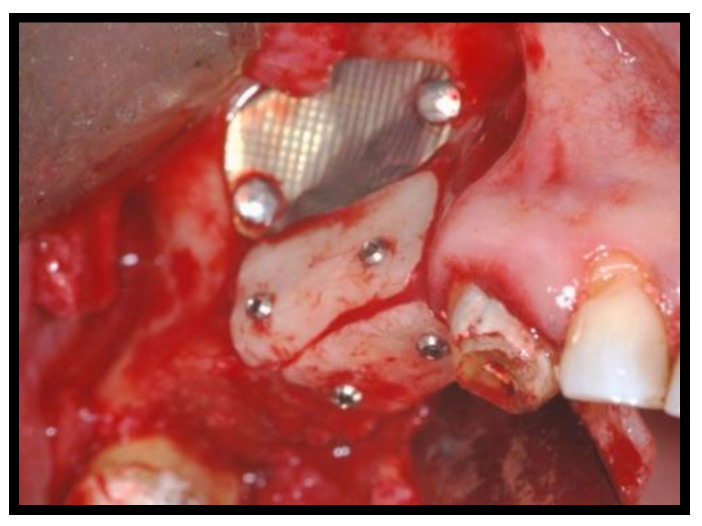
The titanium membrane placement (Group Bio-Oss^®^).

**Figure 9:**
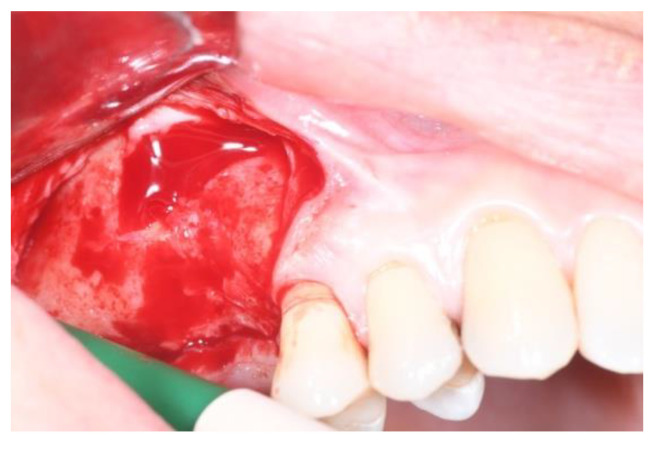
The titanium membrane remotion at four months (Group Algipore^®^).

**Figure 10:**
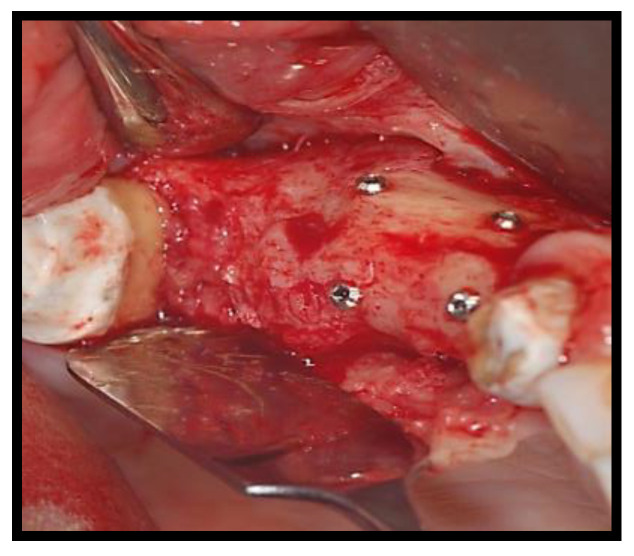
The titanium membrane remotion at four months (Group Bio-Oss^®^).

Titanium membrane (Omnia) was placed (Figure 7 and Figure 8) and removed after ~4 months (Figure 9 and Figure 10). 

**Figure 11:**
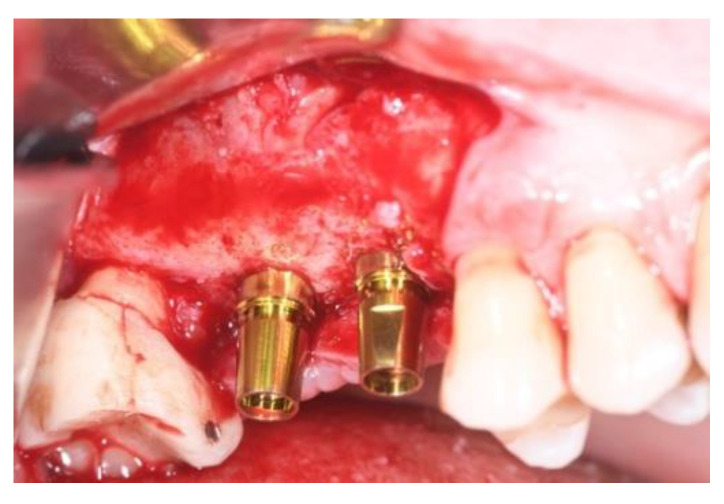
Implants insertion (Group Algipore^®^).

**Figure 12:**
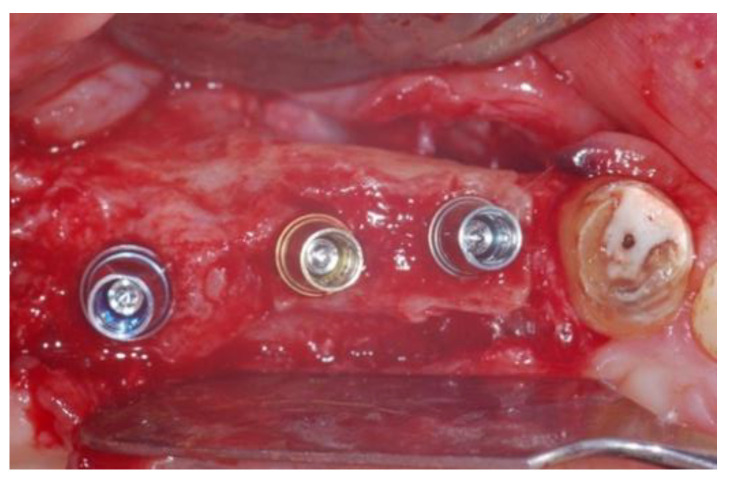
Implants insertion Group (Bio-Oss^®^).

**Figure 13:**
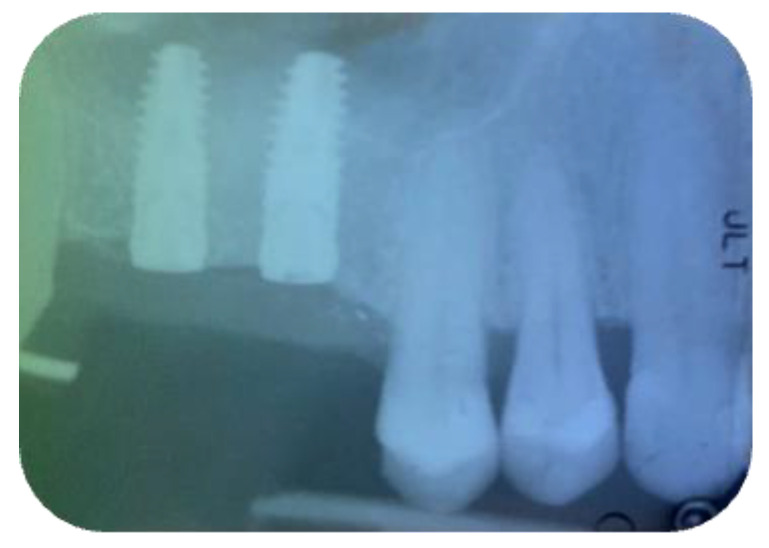
The rx of inserted implants (Group Algipore^®^).

**Figure 14:**
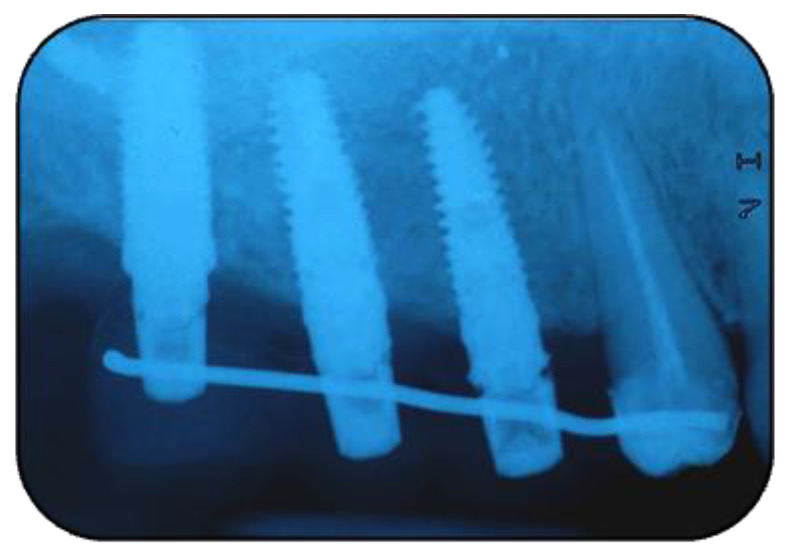
The rx of inserted implants (Group Bio-Oss^®^).

Patients were followed up at 7, 15, 30, 90, and 120 days postoperative. The implants placement was performed after four months (Figure 11, Figure 12, Figure 13 and Figure 14) following sinus floor augmentation. A full-split thickness mucoperiostal flap was raised and the underlying bone crest was exposed for osteotomy. A mid-crestal vertical incision was carried out in order to mobilize a full-thickness flap. The flap was carefully elevated from the palatal/lingual and buccal aspect of the alveolar ridge.

**Figure 15:**
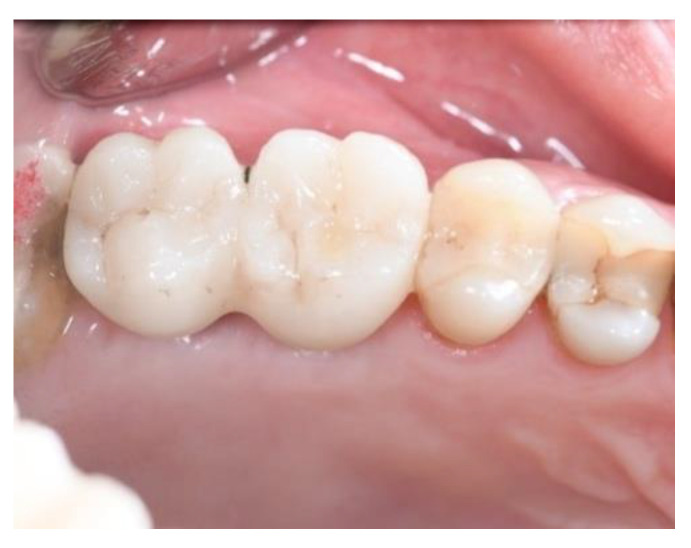
The definitive crowns of implants (Group Algipore^®^).

**Figure 16:**
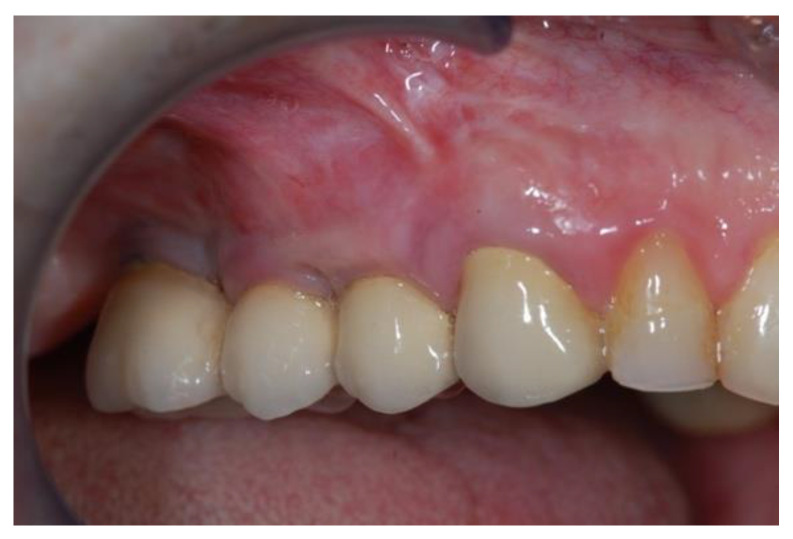
The definitive crowns of implants (Group Bio-Oss^®^).

Ridge expansion was achieved by increasing the diameter of the osteotomes to obtain the appropriate width of bone to better insert the implants. In seven patients, additional trans-crestal sinus elevation was performed on the alveolus by 1.6 mm using Summer’s technique and a 6.0 × 10 mm conical implant was placed. Figure 15 and Figure 16 shown the definitive crowns.

### 2.2. Follow-Up

Patients underwent to maintenance program with half-yearly recalls, that included a full periodontal examination, professional oral hygiene and a clinical/radiographic examination. Absence of symptoms, clinical signs of infection, and progressive marginal bone loss without marked mobility were considered parameters of success. 

## 3. Results

**Figure 17:**
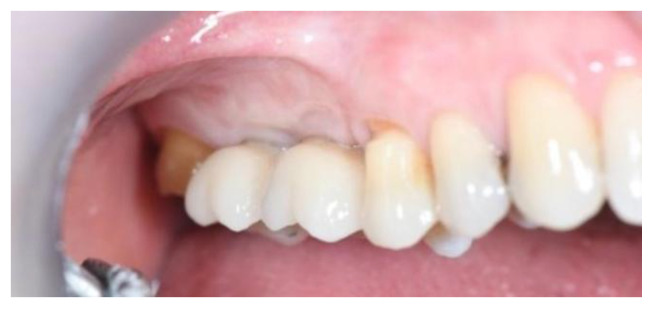
Follow-up at seven years implants placement (Group Algipore^®^).

**Figure 18:**
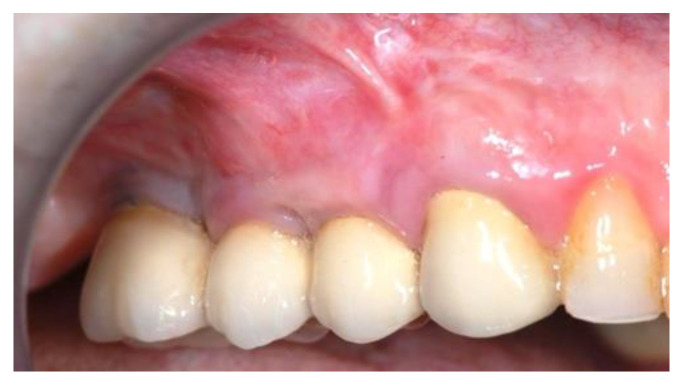
Follow-up at seven years implants placement (Group Control).

**Figure 19:**
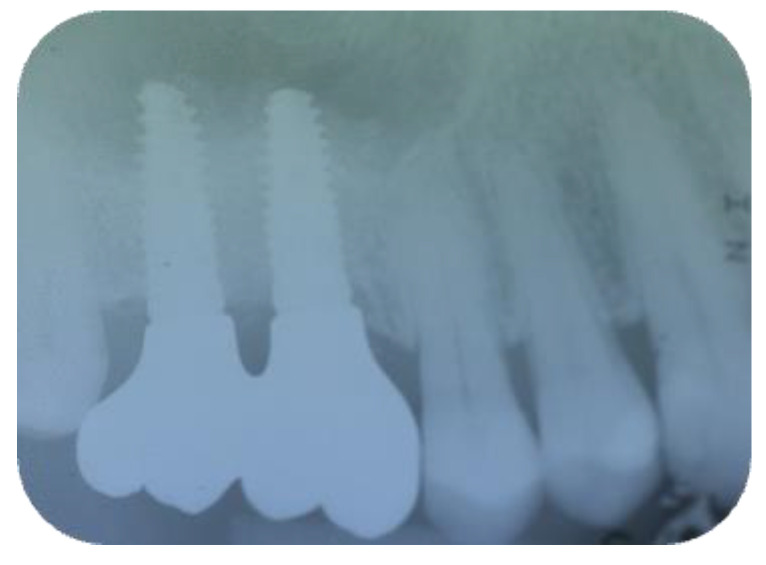
Rx at seven years implants placement (Group Algipore^®^).

**Figure 20:**
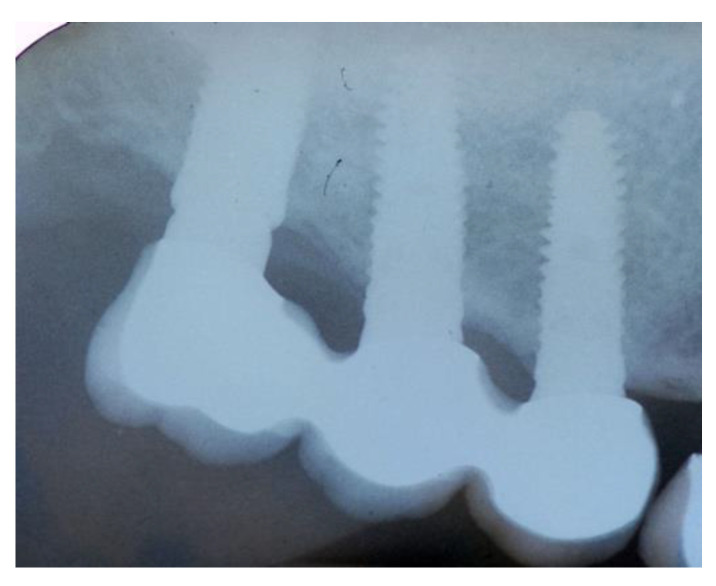
Rx at seven years implants placement (Group Bio-Oss^®^ Group).

The study recruited 57 patients with severe atrophy of upper maxillary crest which needed regenerative surgery approach. The mean age was 47 ± 11.5 years old. No dropouts were registered in this study. The initial opening flap performed with piezo-surgery revealed the presence of a severe bone defect in the vestibular region in thirty-eight cases. The first level orthopantomography followed by a dental scan of 19 patients showed a severe bone loss in the alveolar palatine region. All patients presented a severe maxillary atrophy (crestal height < 5 mm). After the extractions, the severe bone loss and exposition of maxillary sinus membrane was observed. The reopening of the implant site in “second-look” was thus a necessary precondition to have enough bone quantity to proceed and create a predictable positive implant success. The large residual gap was filled with a packing of PRP activated with calcium chloride that allows platelet degranulation in order to obtain PRF (platelet-rich-fibrin) which was mixed with Bio-Oss^®^or Algipore^®^ FRIOS^®^. During surgery, four tubes of blood were harvested and centrifuged and it was obtained 4 mL of PRP by each one. Soft tissue healing was obtained after ~15 days while the OPT images after placement showed radiographic integration and increased peri-implant bone density maintained at the seven years orthopantomography checkup. At four months after the implant placement, radiographic images showed complete osseointegration of the implant (Figure 11, Figure 12, Figure 13 and Figure 14). We recorded perforation of the sinus membrane during sinus lift during two surgeries, without compromising the surgery and subsequent implantation. The lack of osseointegration distinguished by implant mobility and radiological radiolucency were referred to a failing implant. All sinus floor elevations were successful. The orthopantomography checkup show radiographic integration and increased peri-implant bone density maintained at seven years (Figure 17, Figure 18, Figure 19 and Figure 20).

## 4. Discussion

Porous phycogenic hydroxyapatite (PHA) derived from red algae (Algipore^®^), is largely employed as scaffolds in bone regeneration, due to its chemical similarity to bone and interconnected porosity [25]. Bone ingrowth is affected by several mechanical properties of the scaffold, involving the internal porous structure [26]. The pour size influences the permeability and the inadequate dimensions may result in altered bone ingrowth. There are several studies conducted on the fluorohydroxyapatite (FHA) FRIOSs Algipores as a proper biomaterial for the reconstruction of severely atrophic maxillae [27]. Schopper et al. investigated the histomorphological and histomorphometrical examination of 69 trephine specimens who were submitted to maxillary sinus grafting with FRIOSs Algipore [22]. The authors demonstrated that the scaffold elicited generation of new bone in the grafted sinuses, about 23.0% over an observation time of seven months. The findings accord with other authors who found a comparable bone formation after six to seven months, combining porous hydroxyapatite and autogenous bone for sinus grafting [30,31]. Deproteinized bovine bone Bio-Oss is biocompatible and osteoconductive, while is missing of osteoinductive property [32]. Sartori et al. [33] observed a slow but continuous resorption of the Bio-Oss scaffold. These results were contrasting with Schlegel et al. [34] who reported a low resorption capacity of deproteinized bovine bone. The present investigation compared the FHA biomaterial FRIOSs Algipore and deproteinized bovine bone in triggering the formation of new bone in the grafted sinuses of severely atrophic maxillae. We noticed a significant increase in the new bone formation in the areas augmented with both biomaterials. Our findings are in agreement with other authors using a combination of porous hydroxyapatite or deproteinized bovine bone and autogenous bone for sinus grafting [35,36,37,38,39]. 

The maxillary bone in edentulous upper premolars and molars undergoes a remodeling process resulting in horizontal and vertical reduction of crestal dimensions, making it insufficient for implant placement. Sinus elevation via a lateral approach was applied. It is classified as a technique-sensitive procedure due to the high risk of Schneiderian membrane perforation that can occur quite frequently, up to 35%. This procedure offers a positive long-term prognosis and a higher survival rate than the placement of ungrafted maxillary, and in particular, rough-surface implants [40,41,42].

The split bone technique was only performed with very fine chisels as the use of burs could compromise bone preservation. According to Misch and Judy [43], the use of this procedure, with type C bone defect should respect a crestal width between 1.5 and 2.5 mm with a height ranging between 8 and 12 mm. Implant placement, in the ideal prosthetic position, may be compromised by bone resorption due to the presence of an increased interarch distance or an unfavorable horizontal and sagittal intermaxillary relationship [44,45,46]. It was necessary to subject resorbed ridges to regeneration treatment before or concurrently with implant placement in order to increase the amount of hard and soft tissues [47,48,49]. This allowed us to reduce the crown-to-implant ratio, place axial implants, and achieve good occlusion and a quality aesthetic appearance. The alveolar ridge split is a predictable and reliable procedure, characterized by its low invasiveness. This procedure allowed us to achieve significant bone augmentation in the horizontal plane. In the case were we registered vertical bone lost, before to proceed to implantation, we performed bone augmentation according to Khoury’s concept. The bone was collected from retromolar area to reconstructed the vertical defects. Platelet-rich fibrin (PRF) is an autologous platelet concentrate obtained by centrifugation from the patient’s own blood without the use of heparin or anti-coagulants. As with PRP, the PRF procedure is easy, safe, and biocompatible. The results suggested the potential role of PRF in periodontal regeneration and tissue bioengineering as a viable material for bio-graft construction. PRF is a material composed mainly of fibrin membranes enriched with platelets and growth factors optimal for promoting the healing process of hard and soft tissues. Thus, PRF is able to regulate inflammation and stimulate the chemotaxis mechanism. In addition, its gelatinous consistency increases the stability of the clot and graft material. However, being a biomaterial formed directly from the patient’s blood, the amounts that can be obtained may sometimes be very modest. PRF has the distinguishing trait of polymerizing naturally and slowly during centrifugation. The concentrations of active thrombin and fibrinogen contained in PRF are almost within normal physiologic ranges since the material does not require any addition of bovine or humanized thrombin. Fibrin tends to acquire a three-dimensional structure equivalent to the site where it is inserted supporting the healing process [47,48,49,50,51,52,53,54,55]. Aggregation of fibrin monomers leads to the formation of a three-dimensional scaffold, forming a thin mesh of soft, porous graft that allows rapid cell colonization of the wound and surrounding tissues [43,51]. This type of bio-scaffold induces a faster physiological healing process and in combination with bone grafting accelerates the formation of new bone tissue [52]. Derivatives from different species, usually ovines, undergo a series of tests and processes of demineralization, sterilization, freeze-drying. Although widely used xenografts perform similar osteoconductive activity and are relatively cheaper. In addition, their use reduces the need for a second surgery for bone harvesting [53,54,55]. However, xenografts have demonstrated a low ability to induce adequate height and width in large defects, especially those of bovine origin. Few results from histomorphometric analysis showed low resorption rate of transplants after several years revealed residual bovine graft up to 40%, data confirmed by several histo-analyses that reported the same amount of graft detected at three years to that at six months [51,56]. Our study has some limitations. Firstly, the study design with intrinsic restriction; secondly, histological examination is needed to reveal a physiologic framework of bone around the biomaterial particles. Finally, the inclusion criteria were stringent and eliminated interferences due to systemic disease and other factors that may alter recovery. It follows that while the adoption of mimicry approaches has finally yielded positive results, one must consider the interference of multiple variables such as physical, biochemical, metabolic, immunological, and hormonal conditioning. There are huge differences between an inserted bone graft and a mature healthy tissue microenvironment, but even more so there are crucial changes between an inserted graft and the current health status of the recipient [25,27,57]. Consequently, a different intervention should be planned by adapting not only the design of subsequent implants, but emphasizing the treatment plan that fully reflects these differences. Normal healthy adult development occurs in a variety of immunologic, inflammatory, hormonal, and metabolic contexts. The complexity of these factors must necessarily be addressed if the processes are to be united for complete and successful integration of bone grafts and implants [58]. Endocrine signaling gradients that function on a scale of healthy conditions can be subverted in a highly deteriorated situation. Modular implants, including those with smaller units including GFs and cells, can be subjected to the unfavorable internal cellular and molecular microenvironment and eventually can be altered leading to infection, necrosis, and ultimately rejection [43]. The immune-endocrine-metabolic environment that modulates the entire process of regeneration, growth, and remodeling and regulates the influx of cells, molecules, and GFs into growing young and adult bone has yet to be fully elucidated. This is probably a crucial time if we are fully committed to unraveling the potential of evolving bioengineering and regenerative medicine, as immune-endocrine-metabolic factors are significant mediators of bone healing and regrowth or, conversely, can cause a delay in healing if they are suppressed and neglected [59]. This last observation serves to highlight the differences between the developmental processes that occur during normal osteogenesis and those involved in the induction of post-traumatic grafting. Indeed, while inflammation, endocrine imbalances, and metabolic dysfunction may be part of the main drivers of bone decay and graft failure, they are fully functional during normal bone development. The significance of interleukins, cytokines, and hormones in the revascularization, mineralization, and bone/cartilage remodeling activities of hPB-SCs has been profoundly elucidated, and their important role is fully appreciated as external supporters in bone graft therapy [7,9,47]. Our study showed overlapped results over the techniques. However, our findings are not conclusive. The study design and the sample size represent the major limitations of our study. 

## 5. Conclusions

At 7 years follow up, the combination of the particulate autogenous bone with Algipore^®^ FRIOS^®^ or Bio-Oss^®^ showed predictable results over the time in the presence of a small amount of residual bone. However, further studies are needed to confirm the hypothesis.

## Data Availability

Not applicable.
